# Supplementary material for: Comparative genomics provides new insights into the diversity, physiology, and sexuality of the only industrially exploited tremellomycete: Phaffia rhodozyma
Source: BMC Genomics. 2016 Nov 9;17:901. doi: 10.1186/s12864-016-3244-7 (PMC5103461; doi:10.1186/s12864-016-3244-7)
Supplement: Additional file 6: — List of orphan genes with links to PFAM (related to Additional file 1: Table S1). (ZIP 1428 kb) [file 12864_2016_3244_MOESM6_ESM.zip › PFAM_UNIQUE/G03818_P.html]

#                                                                            --- full sequence --- -------------- this domain -------------   hmm coord   ali coord   env coord
  
# target name        accession   tlen query name           accession   qlen   E-value  score  bias   #  of  c-Evalue  i-Evalue  score  bias  from    to  from    to  from    to  acc description of target
  
#------------------- ---------- ----- -------------------- ---------- ----- --------- ------ ----- --- --- --------- --------- ------ ----- ----- ----- ----- ----- ----- ----- ---- ---------------------
  
G03818\_P             -            380 AIB                  PF15334.1    324    0.0094   13.7   0.2   1   1   7.8e-06     0.015   13.0   0.2   191   254   296   364   245   371 0.84 - Aurora kinase A and ninein interacting protein  
G03818\_P             -            380 Amelogenin           PF02948.10   174      0.29   10.4   3.9   1   1    0.0003       0.6    9.4   3.9    38   151    48   159    41   170 0.73 - Amelogenin  
G03818\_P             -            380 SEEEED               PF14797.1    130       6.5    5.6  29.8   1   2       3.1   3.1e+03   -3.1   3.6    91   101    11    21     4    42 0.54 - Serine-rich region of AP3B1, clathrin-adaptor complex  
G03818\_P             -            380 SEEEED               PF14797.1    130       6.5    5.6  29.8   2   2   2.2e-05     0.022   13.6  18.3    14   117   242   349   231   362 0.73 - Serine-rich region of AP3B1, clathrin-adaptor complex
